# Dental Office Efficiency and the Application*Read before the Northern Ohio Dental Society, June, 1920, and published in the November *Summary*.

**Published:** 1921-01

**Authors:** Harry J. Bosworth

**Affiliations:** Chicago, Illinois


### DENTAL OFFICE EFFICIENCY AND THE APPLICATION*

BY HARRY J. BOSWORTH, CHICAGO, ILLINOIS

I have been associated with dentists for thirty years, and, knowing them as I do, have had a wonderful opportunity to make an analysis of some of their conditions. The dentist goes through college, and is told if he does his work to the best of his ability, the fee will take care of itself, but it hasn't in many cases. Some men have paid so much attention to their professional technics and

---

* Read before the Northern Ohio Dental Society, June, 1920, and published in the November *Summary*.

so little to the business side of dentistry that after a time they were living on the bounty of their friends.

Dentistry is a one-man business. You only can produce when you are able to work. When you leave your office you quit earning but your overhead expenses go on. No fee thoroughly understood between the dentist and his patient is too great, provided the patient has been told the truth about the possibilities of his case and a fee has been fixed for the work to be done. If you do not tell him what to expect, later on you possibly must accept his version of the fee he expected to pay which is lower than yours, and then you are in trouble. You can fix a fee for every piece of work, and you can fix it more equitably before you start than after you finish. Most times dentists' figures are never put down until they are put in the ledger, when the entire case is finished, and many times they are changed after they do go into the ledger; and almost always downward, showing the fear that creeps into the dentist's mind when these fees are being arrived at in a hit and miss manner. ,

I can double your income, if I can have you relax long enough to put into action some simple, commonsense things which I will outline, and which are being carried out in other successful lines of endeavor. You build the walls of your own limitations and it may be subconscious to a certain extent, but it is done just the same; and if you wish to improve you must remove those self-imposed walls of limitations. You cannot work your hands much faster, but many times the advice given to a patient is more helpful than the construction work you might do. You need not talk technicalities when going over a case with your patients, it must mean more than that to your patients; it should mean health, so talk health. We have specialists who do not do technical work and yet have all they can do in practice. About seventy per cent. of the effort of the general practitioner is lost motion. I classify it as follows: *Productives are—*

remunerative bridge work, gold inlays, synthetic fillings, lingual bars, pyorrhea and X-rays. *Non-Productives are*— treatments, plates, amalgam fillings, cement fillings, examinations, cleaning teeth, stopping toothache, extractions, anesthetic, broken appointments, etc.

I do not believe dentists know how long it takes to treat teeth, if you did you would handle it as it should be, according to the time used and skill required to accomplish the results. For the fees the average dentist gets for this kind of service the services should be given at the risk and expense of the patient. The tooth is his and not yours, and you should not accept responsibility, except to do the best you can under the circumstances. Contracts for filling teeth needing treatment are charged by the dentist as "treating and filling" for which the dentist will not get his mony until the tooth is filled. Make a fee for the filling, plus treatment, and tell the patient there are several ways of treating a tooth. If patients want to try and save the tooth all very well, but he must do so with a full understanding of the possibilities of failure, and in either event the risk is his. The dentist should be paid for the time given in trying to save the tooth. In the average office this has been wrong, but it can be put on the profit side of the ledger if dentists only realize the time spent, and charge for it. I do not want a dentist to say to his patient that he charges by the hour. It's too much like a factory. But to make an office productive you must find out how many hours you have at your disposal, and how much you must charge per hour to make your income a satisfactory one, and then make your charge accordingly. You can barely figure 1500 hours for a year's work, or six hours per day. I would always say to a patient, "I will do your constructive work for so much, plus treatments." Suppose you do treat and after all the tooth has to come out! Then the burden is back to the owner of the tooth where it belongs.

Many dentists have trouble in collecting. That is often their own fault. If they will only tell a patient what the work will cost before it is started so that he may make provision for the payment, and furthermore tell him the work must be paid for as it progresses, and see that it is paid for by sending out statements on the first of every month for complete or incomplete work, you will have no trouble. Take fewer patients, and be as careful in selecting your patients as they are in selecting you as a dentist. They don't just come to you off the street. They are in most cases referred to you, and as a consequence are engaged when they do come to your office. They are not shoppers then, and if they become so it's because you make them so. Undertake dentistry for what it is, and do not promise too much. Even the very best dentistry may be only temporary and patients should be so advised, so they will not expect too much.

Make a fair profit on every denture dependent on what they cost, and even get a good fee for them. Plates are not productive.

Discourage denture patients rather than promise too much. Tell patients Nature never intended there should be a denture made, but science and skill made it possible. Tell them what your experience with plates has been and that will be discouragement enough. I like to compare dentures with wooden legs. If the leg happens to be off at the right place we can make a fairly good wooden leg, but it is still an artificial member. There are many things a patient can not do with an artificial denture but he is satisfied to have it for what he can do with it. The patient must find out by experience that he must have his co-operation if it's going to be successful, and the best way to get that is to have him learn to wear the denture. If a patient has to replace a plate once in a while, it is no more than he has to do with the other things he is buying and wearing out, or getting tired of.

If an alloy filling is indicated, why shouldn't the fee for it be the same as for gold, unless you are actually selling merchandise? You are dealing in service, based on skill and not merchandise. What would you think of a physician with two prices for an appendicitis operation? Cement fillings are not used much, but when it is indicated why not get a profit fee for time spent on same and disregard the material used?

Examination is the greatest time waster the dentist has. Many times the only reason patients ask you to look over their teeth is that it doesn't cost anything. You don't tell them anything, and they don't pay you anything, so perhaps it is even. But diagnosis is the big thing in dentistry today, and it should be paid for, and the only reason it is not is because you do not ask for it. Take a full set of X-ray pictures and make study models, and then visualize the mouth to the patient, and show your interest in his case. In medicine and surgery today a physical diagnosis means something and is paid for, and in dentistry an opinion of the teeth and mouth conditions by a dentist is worthy of a fee equal to what is charged in medicine and surgery, if it is not, don't give it.

The man who can not get sufficient income out of dentistry usually is the one who has too many patients. The hardest day you sometimes put in, is the day you do not have any real production. The day is coming in dentistry when many men will do nothing but dental diagnosis. Look over the case and have the patient come back for the diagnosis, and then if he wants to go somewhere else for the work, let him go, but there will be a fee for the diagnosis.

Cleaning teeth has, in the past been another complimentary effort. Throw pumice all over the office and get a dollar for it. There was no basis for the dollar charge except it was the smallest sum a dentist could think of. In order to sidestep this grief, tell the patient that to be fair to him and to yourself, the little service you can do for a dollar he can do for himself with a tooth brush. But when he comes to you for real prophylaxis, a work that only a dentist can do, make a proper charge that will show a profit. The biggest thing before a dentist today is prophylaxis, and state laws are going to be enacted to provide for prophylactic nurses to render the service as it is needed. There are not enough dentists to do it, and if the dentists do not put it through the government will do for them.

Do not do anything unless you are paid on a profit basis. Handle whatever charity cases you have to, but be sure the charity is warranted.

Stopping toothache you do not charge for, and yet the nose and throat man charges for all these little services. A lawyer charges not for his time, but for what the service is worth. Suppose you got a toothache at midnight, what would you give to have it stopped? And the public will pay for this service. You won't take advantage of anybody, for they won't let you.

Have a minimum fee per hour, which is your danger point and which must show a fair profit.

Extracting is surgery, and there may be as much responsibility following it as if you cut off a leg. We can not assume the responsibility for less than $5.00 as a minimum fee, and I do not think you have to, as I know of no class who could not pay the fee without a hardship. Extracting is a big possibility, but just think how you belittle the operation. You anesthetize a patient and remove a tooth surgically, is a charge of $5.00 for that too much? You administer the safest and best anesthetic—nitrous oxid and oxygen—and dentists all over the country administer gas and extract a tooth for $3.00. If you should call in a physician to give the anesthetic, he will charge $5.00 and perhaps $10.00. Do not belittle your proposition. This is a responsibility that you haven't a right to assume for a picayunish fee.

"Baby teeth" were put in here at the request of the president of our State Society, who said that in his practice this was one thing that worried him, a custom that had been handed down along with the idea of riding for half-fare. The children are harder to handle than the adults. Why not be paid for handling them? Children's dentistry is a big specialty. Many dentists are now handling nothing but children. It is more preventive than anything else. Dr. Phillip Thomas, of Minneapolis, gives this service by the year, and he usually is paid in advance. Little tots come in twelve times a year for prophylaxis and he is watching their progress, and is doing a tremendous amount of educational work among those children. Yet the average dentist does that work at a loss, because that has been the custom and he follows it.

Many times the older dentists who are giving the very best dental service keep their fees so small that it is difficult for the young man just locating to properly fix his fees so as to show a profit. He feels he must put his fees on the level of the older practitioners. Yet if he only knew it, he could go right on and charge his own fees for his services regardless of the older practitioners.

Dentists let patients break appointments and do not charge for them. In justification they sometimes say there is other work they can do, but they know that is not true for when you have made preparations for a patient and waited many minutes overtime, there is always a great deal of wasted energy before you can take up your other work.

Collect all charges for broken appointments the same as they collect in a theater when you buy a ticket and do not occupy the seat. Tell them that the first charge will be made whether they keep the appointment or not.

Suppose there are several dentists with ability along different lines and patients go to each one of them and there is a similar basis of charges, to some extent there will be a loss of patients to each other. The moment you fear patients are going to leave, you are beaten before you start. You can not handle all the people, there aren't enough dentists to deliver one-tenth of the necessary dental services. If you think so, take a mouth mirror and explorer and go through the dentists' mouths in this room and you will find my contention proven.

I think each man should have one or two of the best lady assistants he can get, and you can get them only by paying the best price. You will not get the best services for a flat price. Give the assistant five per cent. of your improved business in additon to her salary. Then she will help you and save your time. You should have an office that will permit you to finish your work with one patient, so that he can leave the office without coming into contact with the other patients who are waiting.

A good office assistant can pay her own wages and all the overhead of the office, with X-ray pictures alone, and it will make you more efficient in your work too, because when the pictures do not cost you any personal effort you will use more of them.

Put your day on a six-hour basis, and see that you get what you should for that amount of work. Do not take a minimum fee for the whole six hours. The minimum is your danger point only.

Now I have told the patient there will be a charge for examination. I have the clinical examination, the history of the patient, a study model and an X-ray chart. Say I am working on a six dollar an hour basis; that is the minimum, and the other cases where you can charge more than that will bring it up to $10,000 a year. If you do your work my way you will double your income and you will do it with less care. After going over a case with the data before you, estimate the time and make the charge. Put this on the examination sheet and say plus treatments and broken appointments, if any. To your new patients write a letter setting forth your version of it so as to avoid any possible misunderstandings. The first of the month collect for all complete or incomplete work as shown by the books. It makes it much easier to pay. A large bill at one time is hard for anyone to pay, but it is not hard to pay it in installments.

Be sure to get the patient's full name. It makes it easier afterward in your dealings with him. When the vision of the mouth is fresh in your memory, put down in your appointment book the work to be done at the next sitting; that helps you when the case comes to you again and it enables the assistant to get ready and anticipate your needs. Have a time stamp and use it when a patient arrives and when he leaves. This stamp record is handled by the dentist. Put transient patients down in a different colored ink if possible. When the day is over, the assistant puts in this column the amount of time you have worked, and puts down the minimum charge lightly with pencil. Then at the end of the day you spend ten minutes with her and make the permanent charge, which always is sure to be at the minimum fee and in many cases plus.

---

TRIGEMINAL NEURALGIA. Schulte reviews the recent literature on treatment of what the ancient Arabian physicians described as tortura oris, and concludes by commending roentgen irradiation as legitimate after failure with the injection of alcohol; this can be repeated but not continued too long, with an operation on the nerve as the last resource.—*Zentral für Inner Medizin*—Leipzig.

